# A forum on synthetic biology: meet the great challenges with new technology

**DOI:** 10.1093/nsr/nwaa252

**Published:** 2020-10-17

**Authors:** Weijie Zhao

**Affiliations:** NSR news editor based in Beijing

## Abstract

Synthetic biology aims to redesign and reconstruct living systems for understanding life or for useful real-world applications. In the past two decades, scientists have been able to use engineered living systems to produce many kinds of products from bioplastics to drugs, to construct a minimal bacterium with a fully synthetic genome and to store huge amount of information within a cell. And in 2020, when the COVID-19 pandemic swept across the world, the synthetic biology community became one of the major forces to develop effective diagnostic approaches as well as the drugs and vaccines, to rapidly cope with this great challenge with the state-of-the-art technologies in their hands. In this panel discussion held on 3rd August 2020, eleven pioneering synthetic biologists from six countries across four continents gathered to discuss the development trend, challenges and biosafety issues concerning synthetic biology.

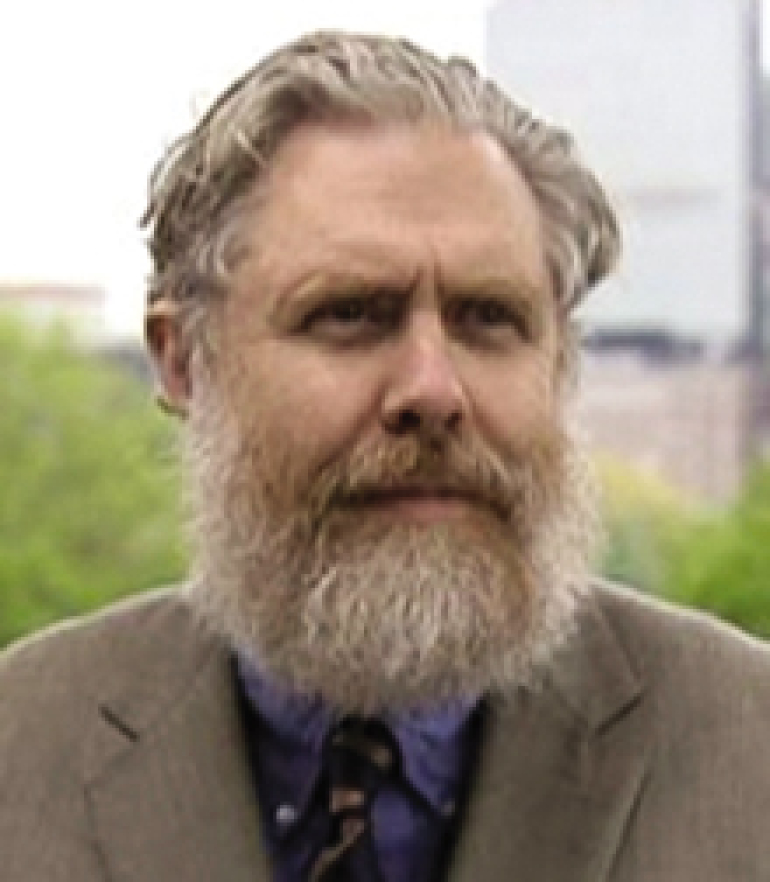

**George Church** Professor of Genetics at Harvard Medical School and Professor of Health Sciences and Technology at Harvard and MIT, USA

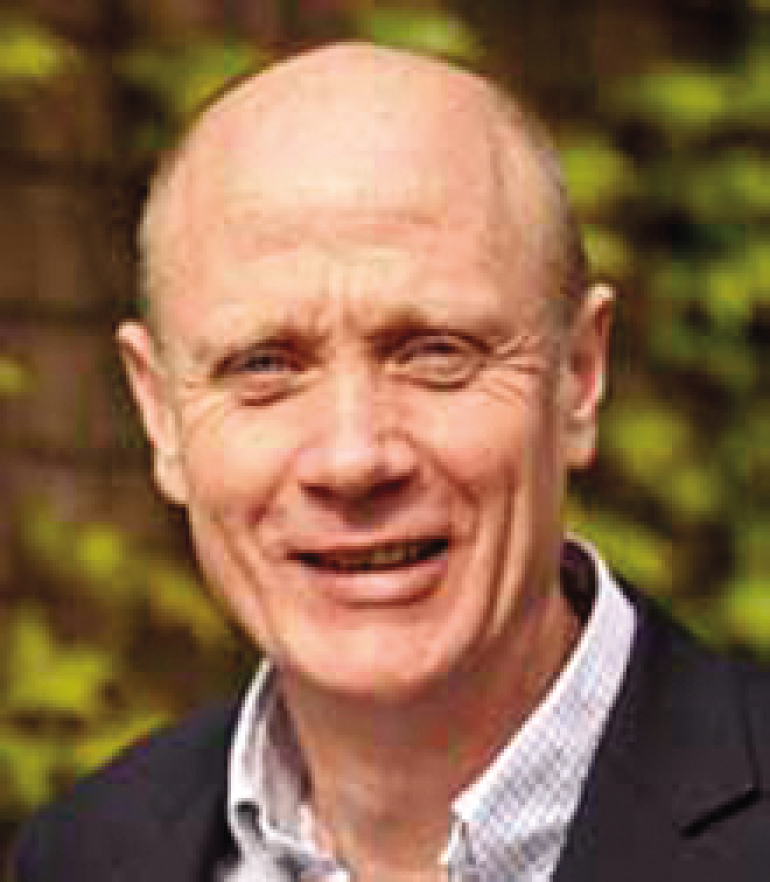

**Paul Freemont** Professor of Structural Biology in the Department of Infectious Disease at Imperial College and a member of the Science Advisory Board of Tierra Biosciences, UK

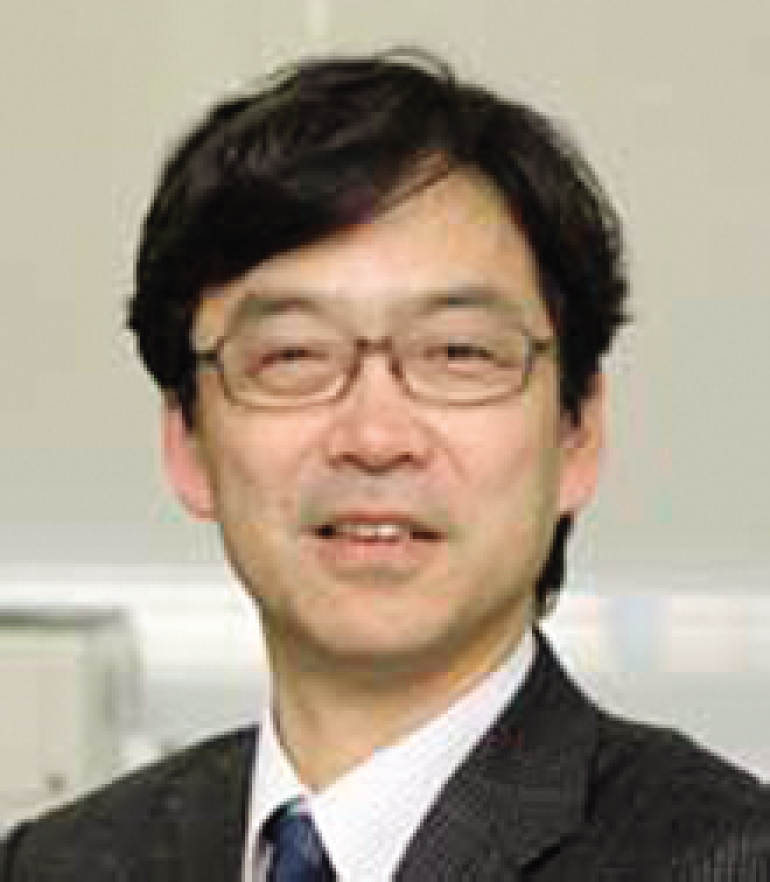

**Akihiko Kondo** Professor in School of Science, Technology and Innovation, and Department of Chemical Science and Engineering at Kobe University, Japan

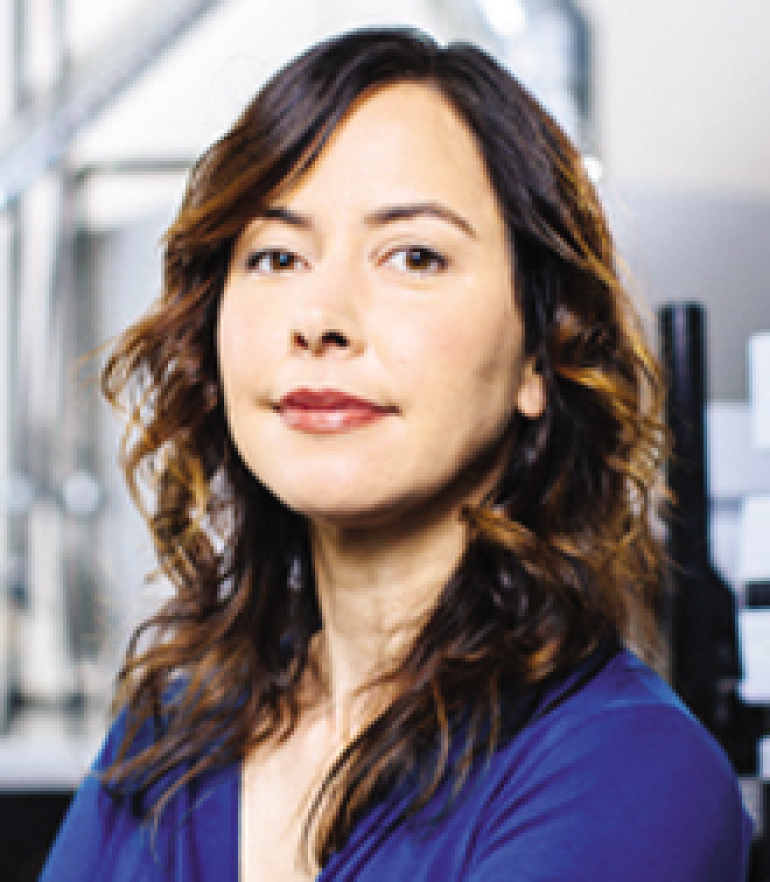

**Christina Smolke** Professor of Bioengineering and of Chemical Engineering at Stanford University and CEO of Antheia Inc., USA

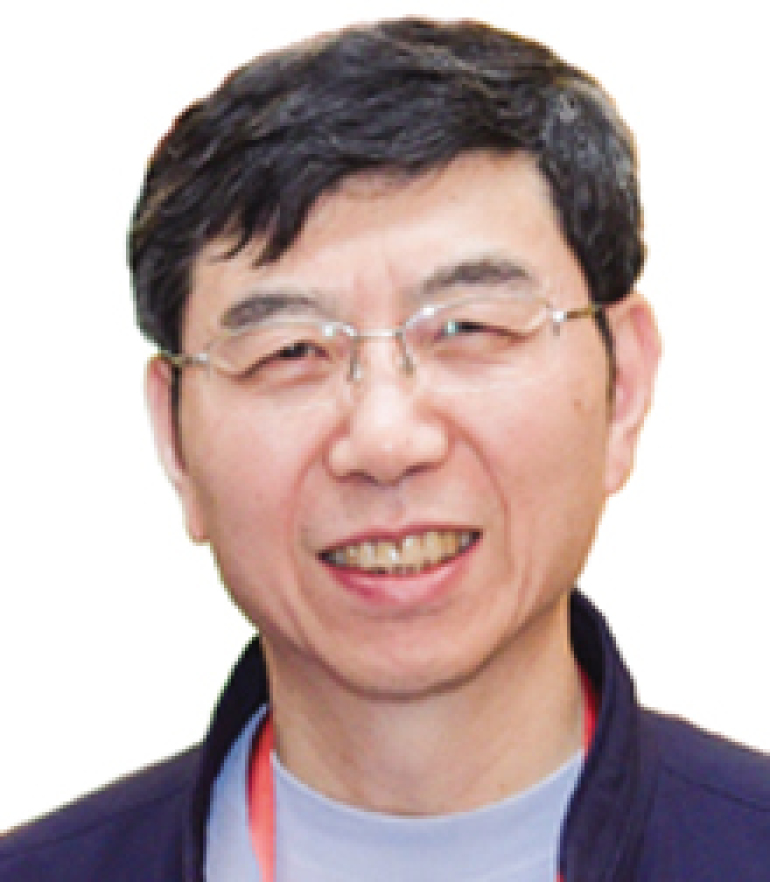

**Xian-En Zhang** Professor at the Institute of Biophysics, Chinese Academy of Sciences, China

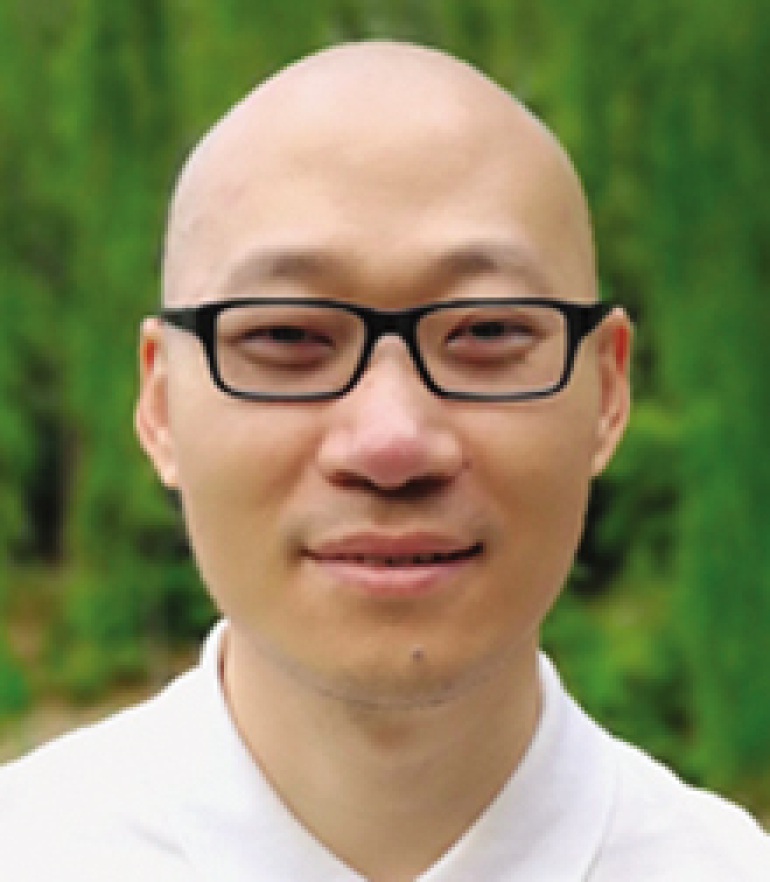

**Chenli Liu (Chair)** Professor and Director of Shenzhen Institute of Synthetic Biology, Shenzhen Institutes of Advanced Technology, Chinese Academy of Sciences, China

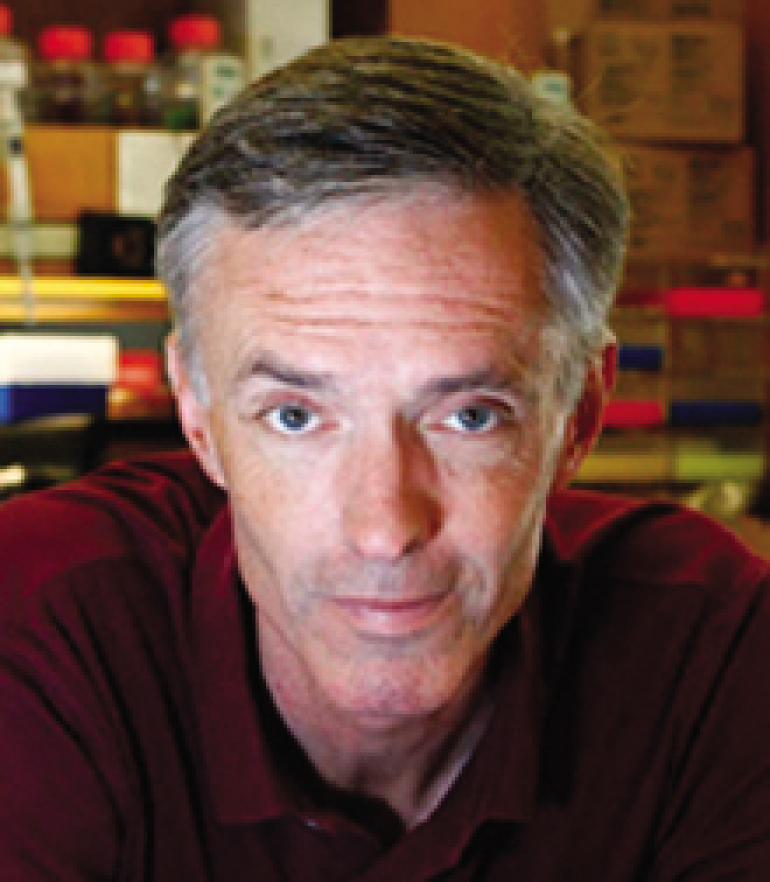

**Jim Collins** Termeer Professor of Medical Engineering & Science and Professor of Biological Engineering at MIT, USA

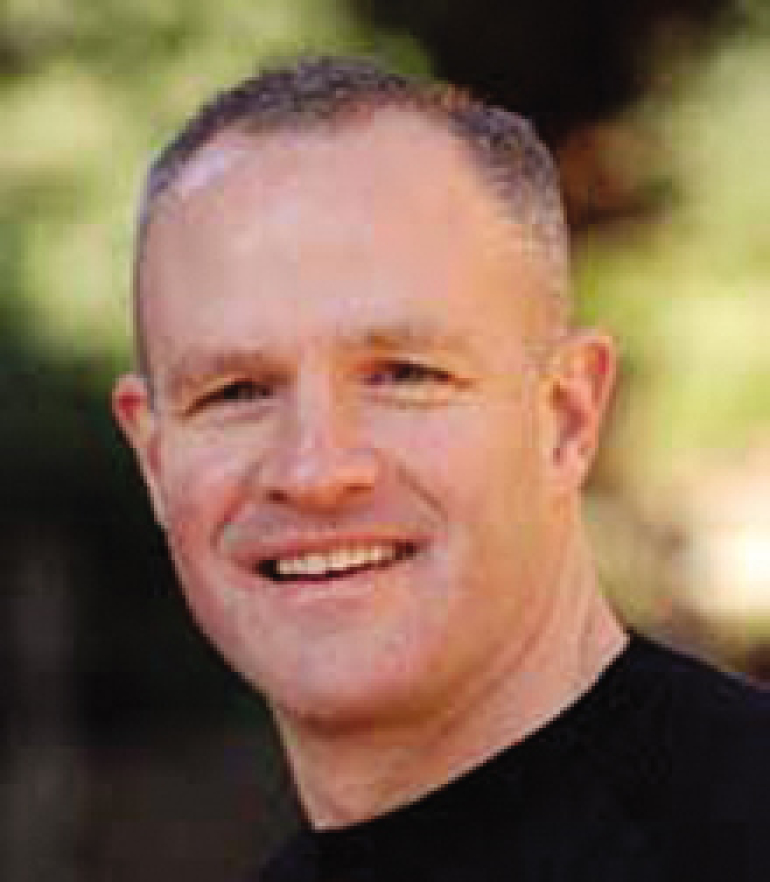

**Jay Keasling** Professor of Chemical Engineering and Bioengineering at the University of California, Berkeley, USA

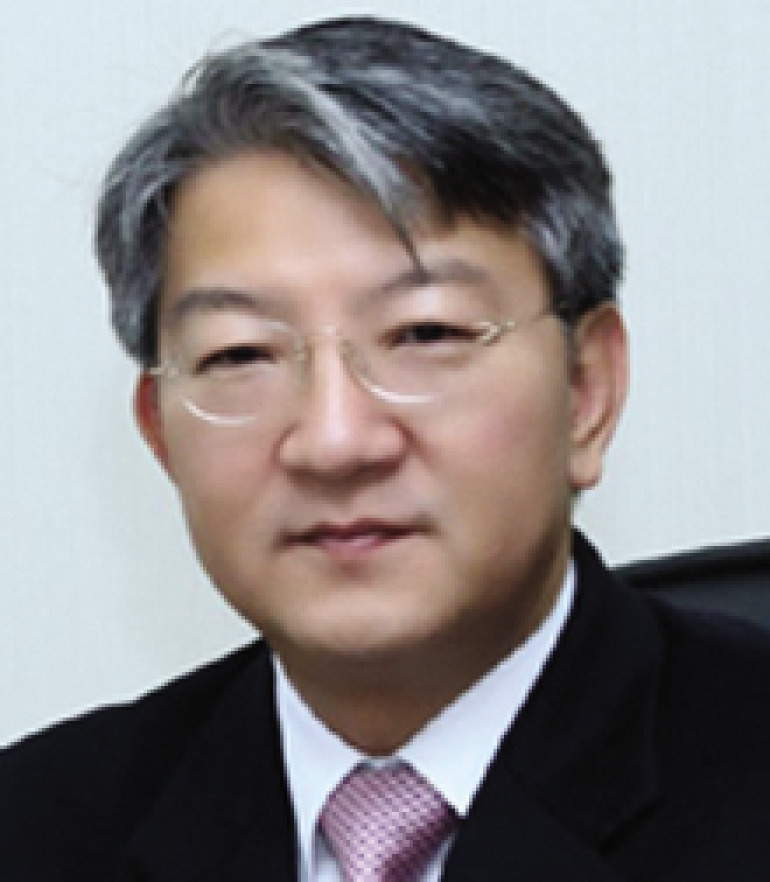

**Sang Yup Lee** Dean of KAIST Institutes and Distinguished Professor in the Department of Chemical and Biomolecular Engineering at Korea Advanced Institute of Science and Technology (KAIST), South Korea

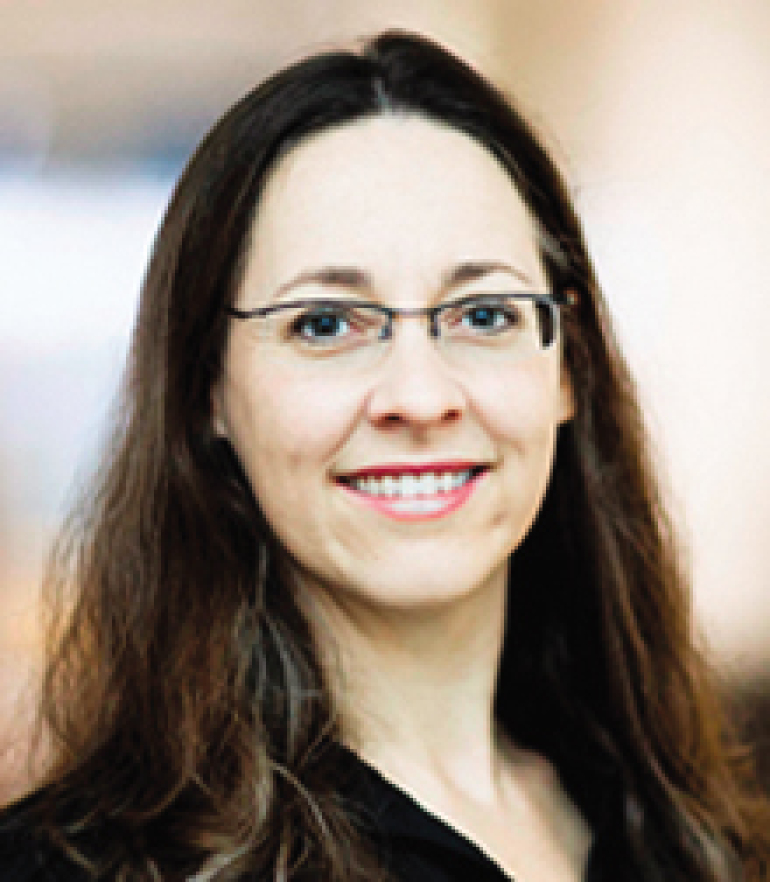

**Claudia Vickers** Director of the Future Science Platform in Synthetic Biology at Commonwealth Science and Industry Research Organization (CSIRO), Australia

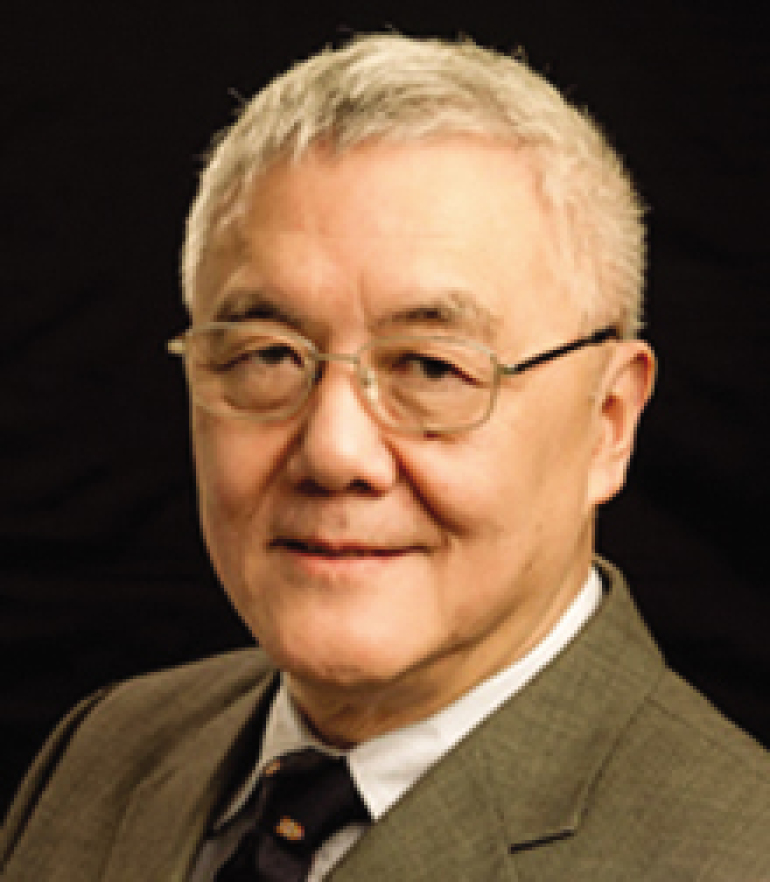

**Guoping Zhao** Professor at the Institute of Plant Physiology and Ecology, Shanghai Institutes for Biological Sciences, Chinese Academy of Sciences, China

**Liu:** Today we have a very distinguished panel from many countries around the world to discuss synthetic biology. The topic is the future of synthetic biology, and how can next innovative revolution led by synthetic biology transform our world. The topic seems kind of topical now. A convergence of engineering and biology is unlocking new research and discoveries that promise advances to cure disease, feed our planet, power our world, extend lives and more. Against this broad background, I’d like to suggest several questions for the panel's consideration.

## THE COVID-19 CRISIS

**Liu:** First, and obviously in the forefront of our minds at the moment, how can synthetic biology be more effective in helping prevent, and deal with, the grand challenges like COVID-19 and other emerging infectious diseases? Maybe we can start from your works on the diagnosis of COVID-19.

**Church:** In my group, we have a number of diagnostic efforts, including home tests that can be done within 15 min. We are also improving the sequencing pipeline using DNA barcoding technology, so that we can test multiple sequence elements of the pathogen and hundreds of thousands of samples at once in a single round of sequencing.

**Collins:** We have multiple efforts using CRISPR and cell-free synthetic biology. We co-founded a company called Sherlock Biosciences, which now has an FDA-approved CRISPR-based COVID-19 diagnostic kit on the market. We are also cooperating with BINX to develop a point-of-care CRISPR-based COVID-19 kit, which is hopefully to be approved by late fall.

Within my lab at MIT, we’re working on two at-home diagnostic efforts. One is a rapid and inexpensive saliva-based test, which could provide a readout within less than an hour. The second is an effort to utilize wearable synthetic biology. We can embed cell-free diagnostic components into a face mask or into inserts that can be added to any face mask, to perform the diagnostic test. We are now finalizing the prototype demonstration of that technology.

**Freemont:** When the crisis hit the UK, there was a real need for urgent testing. At that time, there was an enormous shortage of reagents around the world, so we rapidly repurposed our open platform technologies to become reagent agnostic. We increased the capacity of one platform to do a thousand tests in 12 h. These platforms are quite small, which occupies about 2 m of bench space and can sit in a small lab. Now a major hospital in London is using five of these platforms to carry out over 4000 tests a day. That is part of the Pillar 1 testing in the UK.

**Vickers:** Now I work full time for the Commonwealth Scientific and Industrial Research Organisation (CSIRO), which is Australia's national science research agency. CSIRO works on almost all aspects about COVID-19. We have run projects through our biofoundry and we offered it to support national diagnostics as we’re doing enormous amounts of screening (although it wasn’t needed, fortunately). We are also combining diagnostics and therapeutics to develop sense–response systems, which really minimize the time between identification and treatment and essentially provide more treatment opportunities for the patients.

**Zhao:** My laboratory is working to improve the accuracy and sensitivity of the nucleic acid diagnosis, i.e. to solve the ‘grey zone’ fuzzy readouts of rRT-PCR assays and its related false positive and false negative of the diagnosis. We have two major approaches. First, we are cooperating with BGI (Beijing Genomics Institute) Chip Technology to develop a CRISPR-based fast diagnosis instrument, which is now under the clinical trial evaluation. Second, we are developing a new reagent called Specific Enhancer for PCR-amplified Nucleic Acids (SENA). This method can reduce the limit of detection from more than three copies per PCR reaction down to a little bit more than one copy per reaction.

**Zhang:** My colleagues jointly developed an N protein detection kit for the diagnosis. Asymptomatic infection has become a new concern in the post-pandemic situation. We are paying more attention on the mechanism and thinking about how to identify these patients.

We can embed cell-free diagnostic components into a face mask.

—Jim Collins

**Liu:** Thanks. How is synthetic biology helping to develop COVID-19 therapies?

**Church:** Synthetic organs containing pneumocytes differentiated from human stem cells can be useful for the testing of new therapies. We are now working on that.

**Collins:** In my lab, we are developing short RNAs that can target different regions of the viral genome to knock down its replication. We are also developing a cell-free encapsulated diagnostic system that can detect the presence of the virus while treating.

**Keasling:** We are working on some alternative small molecules for therapies, one of which is a replication terminator for viruses.

**Lee:** We made a fully synthetic human single-chain antibody library with the largely diversified complementarity-determining regions (CDRs). We are screening against several different potential antigens, not only the CDRs or the Spike protein, but also the other regions.

Another approach we are taking is identifying small-molecule drugs. Our lab worked on metabolic engineering and we have established several tools for enzyme design and advanced algorithms for docking simulation. We used the tools to simulate the binding of about 6200 different small molecules with major SARS-CoV-2 proteins, including its major protease and the RNA-dependent RNA polymerase. These 6200 small molecules are approved drugs or potential drugs under clinical trials whose safety has been validated. Interestingly, we identified 52 potential drugs. We are now performing further tests and we hope to find some of the drugs that are much better than the existing ones to treat this virus.

**Liu:** Thank you. What about the vaccines?

**Collins:** On the vaccine front, you may know that Moderna's vaccine is already in the Phase III clinical trial and its key underlying technology of synthetic mRNAs was co-developed by our lab with Derrick Rossi and George Daley about 10 years ago.

Within our lab, we’re taking a much slower approach to engineer a BCG (Bacillus Calmette–Guerin) vaccine, which is an attenuated vaccine that has been used for 100 years to protect against tuberculosis. We are expressing SARS-CoV-2 antigens on its surface so as engineer it to be a COVID-19 vaccine. Now it's in the middle of animal trials. The BCG vaccine has several advantages: it is readily scalable, inexpensive and does not need refrigeration. It would be very well suited for use in developing countries.

**Zhao:** My lab is also working on BCG-based vaccines. Of course, this is based on our previous study of *Mycobacterium tuberculosis* and our current understanding of COVID-19 about its clinic response related to the native immunity.

**Kondo:** COVID-19 is a global disease, so it's pretty important to deliver vaccines to all the countries. So, we are trying to develop some oral vaccine systems, which are easy to use and can be used in the developing countries.

**Freemont:** We are using our biofoundry platforms to collaborate with Robin Shattock, who has been leading the imperial vaccine efforts on self-replicating RNA. This is similar to but not quite the same as the Moderna technology. It's a little replicon encapsulated in a lipid vehicle, which can be put into muscle cells and starts replicating and producing virus Spike proteins to stimulate immune response.

**Keasling:** We’ve been working on adjuvants for vaccines. All vaccines have adjuvants in them that can activate the immune system so that the vaccine can take hold better. We’ve been working with a large pharmaceutical company on small-molecule adjuvants. One really interesting adjuvant is a natural product that comes from a plant. We are taking the genes out of the plant and putting them into yeast so that we can produce this adjuvant much faster. The company told us that we probably need a billion doses of this adjuvant and they’ve been pushing us very hard.

**Kondo:** Besides diagnosis, therapies and vaccines, I think the prevention of infection is also very important. We are working to develop new ways to stimulate our immune system by modulating the microbiome, which may be one of the approaches that can improve our ability to fight the virus.

**Church:** We’ve been advocating the BioWeatherMap Initiative since 2002, which is an environmental sensing effort that aims to clarify the geographical and temporal distribution patterns of microbial life. In the context of COVID-19, people are starting to take it seriously for the first time. With the BioWeatherMap network, we would be able to know the microbial distribution in the local communities. We can detect the patient zero and the pathogen transmission much earlier and the community can act faster. We hope this system can play a stronger role in the future.

**Zhao:** At this moment, we already have more than 80 000 genomic sequences of SARS-CoV-2 all over the world and our center of BioMedicine Big Data has been trying to develop a follow-up system that may enable people to analyze these genetic data in a real-time manner. In addition, by correlating the sequence variation with the corresponding epidemiology data, people may clearly see how this virus is changing in different areas of the world and at different times of the pandemic.

Synthetic biology can help to produce a safe and efficient research system to study these contagious pathogens. For example, currently people are using pseudotyped virus to test different kinds of Spike proteins from different SARS-like or SARS-associated virus sources, wishing to provide more information not only for searching the animal origin of SARS-CoV-2 but also to gather more knowledge for vaccine design.

**Smolke:** I would like to provide one additional layer, which is the supply chain of drugs. In this crisis, the US hospitals have experienced a shortage of both devices and drugs. In the current pharma supply chains, there is a great latency from pharmaceutical factories to hospitals, and additionally from farms to pharmaceutical factories, for the many essential drugs that we make by growing and harvesting medicinal plants. There has been a lot of discussion about making these supply chains more robust and faster response in the USA. Synthetic biology can offer ways for us to rebuild the pharma supply system, which can substantially reduce the latency and act rapidly in the face of global public health crises.

**Vickers:** That's right. In CSIRO, we are also working to build environmental biosense systems, to improve the supply chain and to strengthen the fermentation industry to produce useful chemicals, which have mostly been mentioned by the former speakers.

I think in the face of this unprecedented crisis, the sharing of information and biological resources is something really critical. There is still space to improve, but I am positive because we have seen fantastic progress and everybody is working toward the same goal.

Besides diagnosis, therapies and vaccines, I think the prevention of infection is also very important.

—Akihiko Kondo

## SYNTHETIC BIOLOGY: CHANGES HAPPENING AND WILL HAPPEN

### Changes in the past 5 years

**Liu:** Thank you. Synthetic biology has been fast developing. In your opinion, what are the major changes of this field in the past 5 years?

**Collins:** I have been struck by the fascinating innovations that are emerging from cell-free synthetic biology. These include translational efforts on paper-based diagnostics, rapid prototyping, portable biomolecular manufacturing and even synthetic biology educational kits.

**Freemont:** I agree. There is a sort of mini cell-free revolution going on in our field.

**Kondo:** I think the biggest change in the past 5 years is the introduction of artificial intelligence (AI) and other data-driven approaches, which greatly facilitated our field. These computational design abilities will continue to improve in the future.

**Lee:** The most exciting thing within the past 5 years is probably not just a single technology, but rather the approach of integrating all these great synthetic biology techniques and strategies to improve the design of cellular metabolic and regulatory circuits, and eventually achieve desired cell functions, or to enhance production of desired bioproducts.

**Smolke:** Within the past 5 years, there has been increasing recognition of the importance of synthetic biology. There's been huge investment from the governments and many start-up companies forming to apply this technology across diverse industries, particularly in the USA. In the coming years, we should see the realization of these investments and their successes from a commercialization perspective. We will see lots of applications, including those directed to more complex systems to offer therapies and microbiome applications.

The most exciting thing within the past 5 years is probably not just a single technology, but rather the approach of integrating all these great synthetic biology techniques and strategies to improve the design of cellular metabolic and regulatory circuits.

—Sang Yup Lee

### Challenges

**Liu:** What is the biggest challenge in synthetic biology at the moment?

**Freemont:** When synthetic biology technology was established as a field, one of the great goals was to create a predictable design process that would enable us to design any living system at the genetic level to perform a particular task with robustness and predictability. I think that is not succeeded yet, so one of the biggest challenges for this field is to establish predictable biodesign by the integration of the design and testing cycles and the machine learning and data-driven approaches.

**Kondo:** Another challenge is that we still don’t know much about the complex living systems, the gene functions and their interactions. I think it's pretty important to develop some tools to more rapidly understand complex living systems such as higher organisms and microbiomes.

**Lee:** That's right. How to link gene to mRNA, mRNA to protein, protein to its activity, activity to metabolic flux, and link all of these with regulatory and signaling pathways is a great challenge to me. If we can use big data and deep learning tools to decipher those relations, it will be possible to perform better metabolic engineering, synthetic biology design and also synthesis of any desired products, including small molecular and biological drugs for COVID-19 treatment.

**Vickers:** I agree that the major challenges now are the difficulties to design and to learn about the living systems. We often need to fight against the forces of evolution in order to get designed circuits to work. I hope that the developing technologies including the computational efforts and DNA storage can help us to better understand and design life systems.

One of the biggest challenges for this field is to establish predictable biodesign.

—Paul Freemont

### Trends for the next 5–10 years

**Liu:** What do you predict for the next 5–10 years? What will be the next breakthroughs in synthetic biology?

**Church:** I think organoids, aging reversal technologies and DNA data storage *in vivo* are some of the major things for the future. Better organoids will help the development of therapeutics and now we have new 3D bioprinting ways to build such organoids. DNA data storage *in vivo* is another big growth field. We have stored a terabyte of information in about 30 ng of DNA, which is 1 billionth of a mouse's weight. So, we can store in a cell its own developmental lineage information, as well as any other information you can tap into the transcriptional level.

**Collins:** I think there will be two exciting developments in the next 5 years. One is that there will be a reintroduction or reemphasis of computation. Computation drove the field of synthetic biology in its very early days. Now we are recognizing that we have not understood the design principles as well as we should, so many people are turning to big data and AI approaches to uncover relevant design principles. Second, I think we’ll see an increased utilization of synthetic biology as a tool for expanding our understanding of molecular biology. We can use synthetic biology to explore, probe and understand endogenous biological systems.

**Lee:** I hope that we can finally streamline the entire design and construction processes through the aid of *in silico* systems, which can be applied to many different exciting topics. I’m sure that this would be exciting to see in the future.

**Smolke:** We all understand the importance and potential of AI and data-based techniques. One of the challenges here is to get high-quality rich datasets, so that we can get these tools working effectively to advance design.

**Zhao:** That's right. Additional to the datasets, we also need different kinds of knowledge graphs corresponding to different application scenarios to support the machine learning and other AI approaches to work better.

**Keasling:** About the future applications, I think synthetic biology should be utilized to deal with the major challenges of our planet, including aging and agriculture. Everyone ages, and synthetic biology should help to extend life span and to make life more livable as you get older. In this space, we may start by modulating the immune system to counter age-related autoimmune disorders. Additionally, synthetic biology can be used to engineer microbiomes, which would be a feasible way to deliver therapies and to modify the body environment.

Another huge opportunity is in the food and agriculture space. We need to deliver food to everybody in a way that's good for the environment. We’ve seen a lot of interesting companies getting started in this space, and there will be great progresses.

**Vickers:** Another dramatic challenge of our planet is about the environment and ecology, in which synthetic biology can also help. For example, we recently identified some key genes for the relationship between corals and their related microbiomes that can be used to engineer resistance to coral bleaching. We may use synthetic biology technologies to help these species deal with the changing climate.

Synthetic biology should be utilized to deal with the major challenges of our planet, including aging and agriculture.

—Jay Keasling

### Summary

**Liu:** I would ask our last speaker, Xian-En Zhang, to sum up this part of discussion.

**Zhang:** The major changes in the past 5 years and the coming breakthroughs can be summarized in three aspects. First, build to learn, which is the original concept of synthetic biology. We are exploring new ways to understand evolution, diversity of living systems and disease mechanisms. Second, build to use. We are utilizing synthetic biology for so many applications, from medicine and drugs, fine chemicals and energy, agriculture and food to the protection of environment and ecosystems. Moreover, synthetic biology is intersecting with other disciplines, enabling future biomaterials, biosensing, biobatteries, DNA storage and many other interesting concepts. Also, researchers are interested in the engineering of more complex systems, such as higher organisms and microbiomes. Finally, the research tools are fast developing, including the gene sequencing, synthesis and editing technologies, computational approaches, 3D printing technologies and so on.

About the current challenges, I agree that predictable design is the major one. There are restrictive factors to be overcome before we can better understand and design complex living systems.

Finally, I would like to give a short summary: synthetic biology opens the new era of life sciences and it's revolutionizing the current biotechnology.

Synthetic biology opens the new era of life sciences and it's revolutionizing the current biotechnology.

—Xian-En Zhang

## BIOSAFETY AND ETHICS

### Alleviate misconceptions with positive narratives

**Liu:** There are some misconceptions about synthetic biology among public. What are the common misconceptions people have? How can we combat these misconceptions and communicate more effectively?

**Freemont:** These issues have been around for many years. In the COVID-19 crisis, many people suddenly realized that synthetic biology technologies could construct thousands of different viruses very easily in the biofoundries. So, it's very important for us to develop the positive narratives, to tell the public that there are thousands of natural viruses waiting to come into our communities, and it would be very good to make some of the viruses in the labs and test their infectivity in controlled environment before they actually infect humans, so that we can deal with them better in the future.

We also need to show more successful examples of synthetic biology to the public, so that people can see that this technology is amazing and it can help the broader society in many different ways.

**Zhao:** In China, there are many misconceptions about GMO (genetically modified organism) and gene therapy. These negative voices are major obstacles that should be removed if we want to implement more synthetic biology technologies in agriculture and medication.

**Vickers:** In our Future Science Platform, we have a program called Maximizing Impact that targets the social, ethical, legal, regulation, policy and institutional issues around synthetic biology. Maximizing Impact did a really interesting piece of work that we call the National Baseline Survey of Public Attitudes toward Synthetic Biology, where we interviewed 8000 people across Australia to find their thoughts on synthetic biology. We found that most people had very poor technology recognition. However, the good news is that once we told them a little bit about the technologies, most people were interested in knowing more. And on average, their attitudes to the technologies are more positive than negative. The most important thing is trust—if the public generally trust the scientists and have confidence of the scientific governance, we are better able to communicate with them and alleviate the misconceptions, and also to deliver impact from the science.

I also want to mention that there's a lot of focus in the media around terraforming other planets (like Mars) and things like that, which sounds really exciting. However, I think it's more important to fix the planet that we already live on. We should use synthetic biology as a tool to replace the unsustainable processes that we use currently, and build a better planet for our children and our children's children.

**Smolke:** I think a common misconception is that people often believe that scientists can engineer living systems very easily, which is generally not true. The public does not always understand where we are with the technology and where we are going with it and over what time frame. There are also misconceptions about how robust the engineered systems are, particularly in environments for which we haven’t engineered them for. These details are not very well described in the stories that come out to the public.

People often believe that scientists can engineer living systems very easily, which is generally not true.

—Christina Smolke

### Global standards for ethics are lacking

**Smolke:** Concerning the ethical issues, I think one of the challenges is that we don’t really have a global standard for ethics on what is acceptable and what is not acceptable. Generally, there are different opinions about GMO, germline editing and other things in different countries, and even within the same country. We need to work to establish global consensus about that, because the technologies and their impacts are globalized.

**Zhao:** That's right. As Paul mentioned, the biosecurity and biosafety issues raised in the COVID-19 crisis are indeed very serious. However, I think it's also a good opportunity to get governmental and administrative support for international collaborations to set up standardized principles and regulation rules for synthetic biology. Once the principles and rules are set, everyone should follow the agreed restrictions and any activity breaking the rules should be prohibited.

**Zhang:** Synthetic biology is still young and unexpected ethical issues may come in the future, so the related ethics and law research works should be carried out together with the technological research works. In China's synthetic biology research programs, we have special research projects for the research of related ethics, education and law issues.

**Kondo:** In Japan, we don’t have so many good researchers on ethics. So, I hope that the global researchers can help to create standard textbooks so that we can educate the young scientists about the ethics issues.

**Collins:** Within our engineering department, we have done a very poor job in teaching our students ethics. We basically just throw the ethics problems into the class for discussion, and do not clearly teach or explain the underlying ethical problems needed to address the problems and make informed, thoughtful decisions. I think we as a community need to do a much better job on training our young students to make appropriate ethical decisions in their professional lives.

Once the principles and rules are set, everyone should follow the agreed restrictions and any activity breaking the rules should be prohibited.

—Guoping Zhao

### Ensure biosafety with technologies

**Liu:** As researchers, we need to ensure the safety and security of our technologies. So, how can we prevent the potential problems by technological methods?

**Church:** We developed technologies of genetic isolation, such as the recoding technology, to keep our designed organisms safe to the environment. The designed organisms depend on a nonstandard amino acid that's not found in nature, so that it would be difficult for them to escape. We can also add kill switches to prevent the escape, but this is not always safe if it can be reversed by recombination.

In particular, we can change the genetic code of a cell, and by this relatively simple trick, the cell would be systematically resistant to all viruses.

**Keasling:** There are a lot of interesting efforts in George's lab, including the recoded genetic systems and the kill switches. I think it's important to make the designed organisms more stable, so that they can perform the designed function for a long period of time and do not mutate in the environment.

**Lee:** There is a major international activity last year proposed by World Economic Forum and Nuclear Threat Initiative, which aims to develop a standardized DNA sequence screening mechanism. It will be a fully automated and low-cost mechanism based on the global big data to screen out any suspicious DNAs that might be harmful to humans or the ecosystem. This plan has not kicked off in full scale because of the COVID-19 pandemic, but I think we do need such a system to help prevent the misuse of synthetic DNA. The system itself will not solve all the problems, but it can help to minimize the risk we might have.

**Kondo:** It's my first time to hear about this program and I think we do need such a software or algorithm that can automatically evaluate the safety of the gene circuits. Furthermore, we also need to develop a perfect virus containment strategy to prevent large-scale environmental problems.

We developed technologies of genetic isolation, such as the recoding technology, to keep our designed organisms safe to the environment.

—George Church

## RUN THE INTERNATIONAL ORGANIZATIONS

**Liu:** There are some international synthetic biology organizations and programs, like GP-write, GBA and EBRC. How are these programs coherently organized? How do they advance synthetic biology? Are there any difficulties?

**Freemont:** The GBA, the Global Biofoundries Alliance, was launched in May 2019 in Japan. At the moment, GBA has 29 institutions that have signed a memorandum of understanding. The aim of the GBA is to bring the idea of biofoundries to everyone's attention, to promote their activities, to promote sharing of protocols and open source technologies, and to promote the whole idea of high-throughput design.

It has several working groups. Some of them are quite active, such as the software working group and the grand challenge working group. Currently, one of our major goals is to develop protocols around COVID-19 testing, as well as rapid vaccine and antigen production. Now two major continents, South America and Africa, don’t have any biofoundry institution. GBA is working closely with those continents to try to address that issue and thus enhance the equality of technology access in some ways.

For community-based international organizations like GBA, there are two major challenges. First, it's very difficult to find international funding for these collaborations. Second, it's not easy to keep the momentum going, to keep people engaged and make sure that they feel it is worthwhile to put efforts into the joint international programs.

**Church:** GP-write (Genome Project-write) is an international research project started in 2016. About a hundred laboratories worldwide self-identify themselves as part of GP-write, and quite a few of them are commercial. Even before GP-write, we had Synthetic Yeast and *E. coli* Genome Projects, which aimed to resynthesize and remanufacture genomes in many ways.

GP-write works to develop new technologies and to set open standards, which can make industrial approaches as well as the reading, writing and testing of genomes more efficient. In this sense, many GP-write laboratories overlap a little bit with biofoundries. We are also looking for safer ways of genome writing, using multiple techniques to prevent the modified genetic materials from exchanging with the environment.

**Keasling:** Let me talk a bit about EBRC, the Engineering Biology Research Consortium. The EBRC grew out of the 10-year National Science Foundation-funded Synthetic Biology Engineering Research Center (Synberc). Now EBRC has affiliates around the world and is funded by many organizations, including the federal government, many companies and some private nonprofit foundations. We set up a nonprofit in the USA to run the organization. We recruited Douglas Friedman as our executive director, who did a really great job in both getting the organization running and also diversifying its portfolio. There are many challenges in running an organization, but Douglas has really done an excellent job and I am very proud of the development of the EBRC.

The major work of the EBRC is slightly different from GP-write and GBA. Some countries, such as Britain and China, have national-level roadmaps for synthetic biology, but the USA had no roadmap. So, a major goal of EBRC is technical roadmapping. We have developed a synthetic biology roadmap, which is quite technical, and we are now working on the focused roadmaps in areas of materials, microbiomes, and safety and security.

These international organizations always need strong leadership, solid strategy, policy support and long-term funding.

—Claudia Vickers

**Vickers:** These international organizations always need strong leadership, solid strategy, policy support and long-term funding. In Australia, CSIRO provides long-term funding for the Synthetic Biology Future Science Platform. We hope this platform can help with the development of synthetic biology technologies and also serve the society.

**Zhang:** A number of Chinese institutions are members of these organizations, and I do appreciate their efforts to share protocols, which may promote the development of the entire field.

**Kondo:** These organizations played a major role in data sharing and the standardization of biofoundries. This is really helpful for every biofoundry in every country. I also hope that these organizations can contribute more to solve the global grand challenges, which are challenges of the whole planet, but not of a single country.

**Collins:** Such organizations have played a very important role in our field for the last 15 years. I think our community is now in a strong position to consider creating an international society for synthetic biology, one which could be an umbrella organization for our growing community and help to coordinate all the efforts for a stronger future of our field.

Concluding, I think this was a robust and exciting discussion. Our field is still young and is rapidly maturing. We are in a great position to impact many important problems that our world is facing over the coming decades: problems of health, food, water, energy and environment. I think it's an easy prediction to make that synthetic biology will be one of the defining technologies of the century. We have got a lot of work to do. Thank you.

**Liu:** Let me conclude by thanking all of our members of the panel for really excellent insights onto the questions. Thank you very much for this very successful and I believe historic forum.

